# Surgical management of a large calcifying epithelial odontogenic tumor in the maxilla: A case report

**DOI:** 10.1016/j.ijscr.2019.03.055

**Published:** 2019-04-05

**Authors:** Kleber Gruber, Silas Antonio Juvencio de Freitas Filho, Letícia Copatti Dogenski, Ana Carolina da Silva Bocassanta, Luiz Renato Paranhos, João Paulo de Carli

**Affiliations:** a Department of Oral and Maxillofacial Surgery, Graduation in Dentistry, Paranaense University, Cascavel, PR, Brazil; b Post-Graduate Program in Applied Dental Sciences (Oral Pathology), Bauru School of Dentistry, University of São Paulo, Bauru, SP, Brazil; c Department of Stomatology and Prosthodontics, University of Passo Fundo, Passo Fundo, RS, Brazil; d Department of Preventive and Community Dentistry, School of Dentistry, Federal University of Uberlândia, Av. Pará, 1720, Bloco 2G, sala 1, Umuarama, 38405320 Uberlândia, MG, Brazil

**Keywords:** Calcifying epithelial odontogenic tumor, Odontogenic tumors, Pindborg tumor, Surgical treatment, Oral diagnosis

## Abstract

**Introduction:**

The calcifying epithelial odontogenic tumor (CEOT), also known as Pindborg tumor, is a locally invasive benign neoplasm. Histogenesis is controversial.

**Presentation of case:**

A 26-year-old male presented asymptomatic swelling on the right side of the face, with approximately six months of evolution. At intraoral examination, a hard nodule localized in the right posterior region of the maxilla, measuring approximately 5.0 x 3.0 cm was observed. The computed tomography images showed hypodense lesion with points of calcification associated with two non-erupted teeth. We suspect of odontogenic lesions. The final diagnosis of CEOT was established based on the histopathological aspects.

**Discussion:**

This tumor is more frequent in adult men, usually develops in the posterior region of the mandible and may present clinical-pathological similarities with others odontogenic lesions. The radiological aspects observed in the CEOT are variable and depend on the time of evolution of the tumor. The histopathological examination is mandatory to establish the final diagnosis.

**Conclusion:**

Our case was treated with simple enucleation without signs of recurrence in five years of follow-up. Further studies are needed to understand the aetiology and the biological behaviour of this tumor.

## Highlights

• A 26-year-old male presented asymptomatic swelling on the right side of the face.
• The two biopsies (incisional and excisional) were compatible with calcifying epithelial odontogenic tumor (CEOT).
• This CEOT was treated with enucleation without safety margins and showed no signs of recurrence in five years of follow-up.

## 1 Introduction

The calcifying epithelial odontogenic tumor (CEOT) was described in 1955 by Jens Jorgen Pindborg, a Dutch pathologist [1,2]. The origin of this tumor is controversial, and it is believed that it can be derived from oral epithelium, reduced enamel epithelium, intermediate stratum or even dental lamina remnants [1,2].

In 90% of the cases, the CEOT is intraosseous and extraosseous lesions are common in the anterior gingival region [3]. This tumor usually shows a slow and asymptomatic growth and has been more frequent in the posterior region of the mandible [1,4].

The histopathological characteristics are composed of three elements: epithelium, amyloid and calcifications [5,6]. In addition, histopathological variants of this tumor have been reported [1,6,7]. Basically, the treatment is enucleation and monitoring is essential because of the possibility of recurrence around 15% [1,3,4].

The aim of this report was to describe the clinical, radiological and histological findings of a case of CEOT that occurred in the right posterior region of the maxilla in a young adult male. The lesion was enucleated and the case has been followed for five years.

This case report was written in accordance with the SCARE guidelines [8].

## 2 Presentation of case

A 26-year-old man was attended at the Clinical Diagnosis of the Paranaense University (UNIPAR) with the complaint of asymptomatic swelling on the right side of the face, with approximately six months of evolution. Her past medical history was unremarkable.

**Fig. 1:**
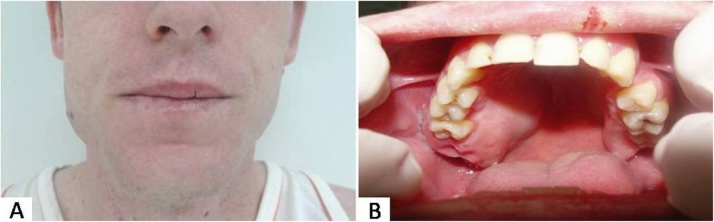
Extraoral clinical appearance showing swelling of the right side of the maxilla (A); Intraoral appearance evidencing swelling in the posterior region of the right maxilla (B).

Extraoral examination revealed discrete swelling, hard and fixed in the right side of the posterior region of the maxilla, causing facial asymmetry, with the absence of lymphadenopathy (Fig. 1A). Intraorally, the swelling was represented by a nodular mass localized in the region of the second right upper premolar to the tuberosity extending in the vestibular-palatine direction, of hard consistency and measuring approximately 5.0 × 3.0 cm. The region was covered by normal oral mucosa (Fig. 1B). Sensitivity and motor function were preserved.

**Fig. 2:**
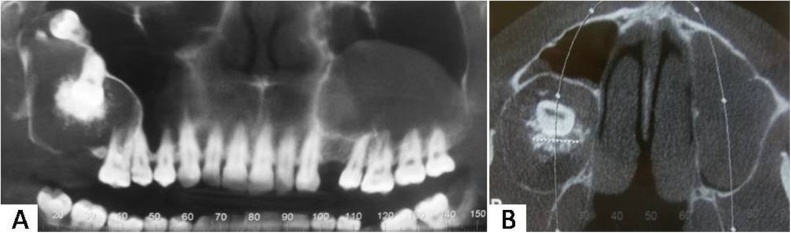
Coronal (A) and axial (B) computed tomography images.

The radiographic evaluation revealed a radiolucent area related to tooth 17, in addition to expansion and reabsorption of the maxillary vestibular-palatine cortical bone with dislocation of the tooth 18 to the floor of the right orbit. Root resorption of the adjacent teeth was not observed. The coronal computed tomography (CT) shows hypodense lesion extending from the second upper right premolar to the orbit base, with structures compatible with teeth in its interior and some hyperdensing points suggestive of calcification (Fig. 2A). In addition, opacification of the left maxillary sinus was observed (Fig. 2A and B). The axial CT image reveals similar characteristics previously described (Fig. 2B).

The clinical hypotheses of diagnosis were the dentigerous cyst, calcifying odontogenic cyst and CEOT. Fine needle aspiration was negative for fluid. The patient underwent an incisional biopsy. The histopathological analysis of the specimen was compatible with CEOT.

**Fig. 3:**
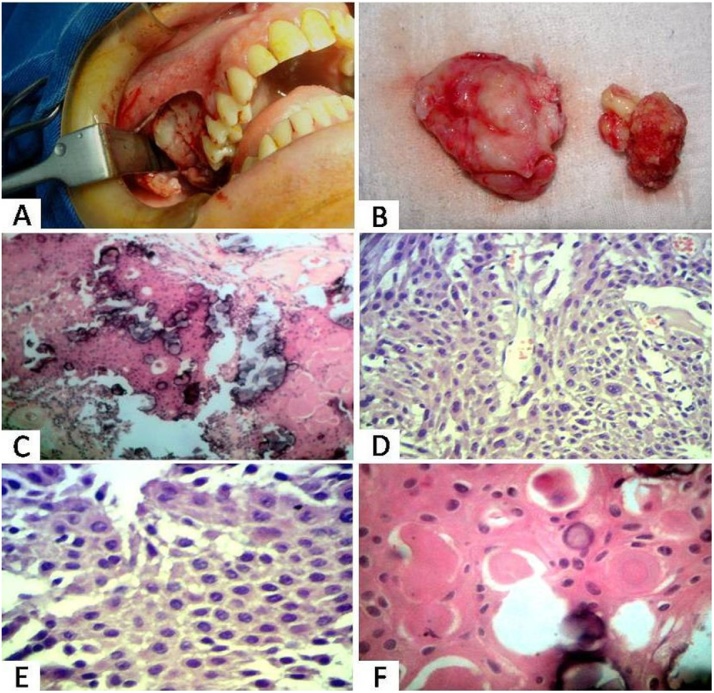
Trans-surgical operative aspect showing access to lesion (A); Surgical specimen of the lesion (B); Histopathological examination (hematoxilin-eosin stain, C–F): Islands and sheets of polyhedral cells, areas of extracellular and amyloid-like eosinophilic material, and small irregular foci of calcification (40×, C); Nests of polyhedral cells forming intercellular bridges and nuclear pleomorphism (100×, 200×, D–E, respectively); Epithelial cells with nuclear pleomorphism interspersed by homogeneous eosinophilic material and concentric calcifications (Liesegang rings) (200×, F).

The patient underwent enucleation of the lesion under general anesthesia and during the trans-surgical procedure, we detected that the lesion was easily detached from the adjacent bone. The surgical access revealed rupture of the vestibular cortical bone (Fig. 3A). On gross examination we observed two fragments of smooth tissue associated with teeth, measuring approximately 6.0 × 4.5 × 2.5 cm, which were sent for histopathological examination (Fig. 3B).

Microscopically, the tumor was composed of islands and sheets of polyhedral cells forming intercellular bridges, areas of extracellular and amyloid-like eosinophilic material, small irregular foci of concentric calcifications (Liesegang rings) and cells with nuclear pleomorphism (Fig. 3C–F). Based on these microscopic characteristics, the final diagnosis of CEOT was established.

The patient has five years follow-up and there were no signs of recurrence.

## 3 Discussion

In the 4th edition of the World Health Organization Classification of Head and Neck Tumors, 23 odontogenic tumors have been listed and CEOT remains among benign epithelial OT subtypes [9].

The odontogenic tumors represent between 2.3% and 4.7% of all lesions diagnosed in specific anatomic pathology services of the buccomaxillofacial complex [10,11]. The CEOT is also known as Pindborg tumor and is considered a rare pathological entity representing less than 2.5% of all odontogenic tumors [[11], [12], [13]], although a prevalence up to 15% has been found [14]. This tumor is more frequent in men [11,13,14] and usually develops in the posterior region of the mandible [11,12,14]. The lesions in the posterior maxilla region, as in the present case, represent less than 25% of all CEOTs [5,11]. Moreover, this tumor is more frequent in individuals between the third and sixth decade of life [12,14].

According to the literature [1,4] and as shown in our case, the CEOT is locally aggressive and non-erupted molar teeth may be associated with the lesion, showing clinical-pathological similarities with dentigerous cyst, calcifying odontogenic tumor and calcifying odontogenic cyst. The slow and asymptomatic growth reported by our patient is also a clinical characteristic observed in patients with CEOT [2].

The radiological aspects observed in CEOT are variable and depend on the time of evolution of the lesions, being able to present from a unilocular radiolucent lesion to an entirely radiopaque mass [2]. In our case, the lesion was predominantly radiolucent/hypodense with few spots of irregular calcifications grouped in close proximity to one of the teeth.

Microscopically, CEOT shows typical characteristics as polyhedral epithelial cells with intercellular bridges, arranged in islands, sheets or cords, with eosinophilic cytoplasm and varying degrees of nuclear pleomorphism, interposed by areas of amyloid material and calcifications (Liesegang rings). In addition, mitotic figures are unusual. It is possible to find variations in the distribution and proportion of the elements that compose this tumor [2,5]. Furthermore, it has been reported that younger cases have more of the epithelial component and older cases are rich in amyloid or calcifications [5]. Approximately one-third of cases of CEOT show clear cells [5] and it has been reported that this histopathological variant is more aggressive, however, remains controversial [2,[5], [6], [7]]. Other variants of CEOT with cementum and bone-like materials and noncalcifying CEOT with Langerhans cells have been described [2].

In general, the treatment for CEOT is surgical enucleation, but the treatment plan may vary among patients [1,7]. It has been recommended to perform curettage or margin of safety with clinically healthy bone removal mainly for mandibular lesions [1,3,7]. Although locally invasive, in the present case we opted for a conservative treatment since the tumor was easily excised and our patient was young. The minimum time of five years of follow-up has been recommended, limited to the few studies of case series due to the rarity of this tumor [1,3]. In the present case, our patient was followed up for this time and showed no signs of recurrence.

In summary, we present a case of CEOT in a young adult patient with clinical-radiological characteristics similar to other odontogenic lesions, and histopathological examination was essential to establish the final diagnosis. Our case was treated with simple enucleation without safety margins due to the location of the tumor, trans-surgical aspects and age of the patient, and showed no signs of recurrence in five years of follow-up. In addition, case series studies are needed to better understand the origin and variations in the biological behavior of this tumor.

## Conflicts of interest

The authors declare that they have no conflicts of interest.

## Sources of funding

There are no sponsors involved in the study.

## Ethical approval

Ethical approval was not required and patient identifying knowledge was not presented in this report. Ethical approval is waived.

## Consent

The written informed consent was obtained from the patient for publication of this case report and accompanying images. A copy of the written consent is available for review on request.

## Author’s contribution

All authors contributed signiﬁcantly and in agreement with the content of the article. K. Gruber, L. C. Dogenski and A. C. da Silva Bocassanta participated conducting the patient. S.A.J. de Freitas Filho, L. R. Paranhos and J. P. de Carli participated in the process of writing of the paper. All authors have read and approved the final version of the manuscript.

## Registration of research studies

Not applicable.

## Guarantor

Dr. Luiz Renato Paranhos.

## Provenance and peer review

Not commissioned, externally peer-reviewed.
